# Myeloid and Mast Cell Progenitors Are Elevated in Atopic Dermatitis

**DOI:** 10.1016/j.xjidi.2025.100390

**Published:** 2025-06-20

**Authors:** Katie Ridge, Barry Moran, Mark A. Little, Cliona O’Farrelly, Jean Dunne, Julianne Clowry, Clodagh Loftus, Conor M. Finlay, Alan D. Irvine, Niall Conlon

**Affiliations:** 1 UCARE Centre, Clinical and Diagnostic Immunology, St. James’s Hospital, Dublin, Ireland; 2 Clinical Medicine, School of Medicine, Trinity College Dublin, Dublin, Ireland; 3 Trinity Biomedical Sciences Institute, School of Biochemistry and Immunology, Trinity College Dublin, Dublin, Ireland; 4 Trinity Kidney Centre, Trinity Translational Medicine Institute, School of Medicine, Trinity College Dublin, Dublin, Ireland; 5 Dermatology, St. James’s Hospital, Dublin, Ireland

**Keywords:** Atopic dermatitis, Immunology, Mast cells, Myeloid cells, Translational medicine

## Abstract

Chronic spontaneous urticaria and atopic dermatitis (AD) are chronic skin disorders characterized by itch. Although mast cells play an integral role in the pathogenesis of chronic spontaneous urticaria, their role in AD is unclear, having a contributory role in a disease largely driven by T helper 2 polarization. Despite this, the role of mast cells in AD is important, given their release of proinflammatory mediators. Recently, myeloid and mast cell progenitors were identified as potential biomarkers for treatment response in chronic spontaneous urticaria. These Lin^−^CD117^+^CD34^+^FceRI^+^ cells appear to have increased egress from the bone marrow in atopy. We measured Lin^−^CD117^+^CD34^+^FceRI^+^ cells in the peripheral blood of 10 individuals with AD and 10 healthy controls. Flow cytometry revealed a significant increase in myeloid progenitors in participants with AD (*P* = .0067). Total serum IgE levels did not correlate with myeloid progenitors. To our knowledge, examination of this cell type in AD is previously unreported. Our findings suggest increased progenitor egress from the bone marrow in these patients and a possible role for myeloid progenitors in disease pathogenesis.

## Introduction

Atopic dermatitis (AD) is a common inflammatory skin disorder characterized by recurrent eczematous lesions with the dominant symptom of intense itch (Langan et al*,* 2020). Whereas the role of T-cell–mediated type 2 inflammation is integral to the pathogenesis of AD, mast cells and the inflammatory mediators they release may have an important role. Mast cells secrete effector cytokines with critical functions in AD pathogenesis such as IL-4 and IL-13 (Voss et al, 2021). Furthermore, the close association of mast cells with sensory neurons in the skin may provoke inflammation and sensation of itch (Voss et al, 2021).

Mast cells are challenging to study given their absence from peripheral blood. Lineage^−^CD34^hi^CD117^int/hi^FceR1^+^ cells in the blood display mast cell–like morphology and gene expression (Dahlin et al, 2016). Mast cell progenitors have been found to be increased in allergic asthma, allergic rhinitis, and chronic spontaneous urticaria (Alvarado-Vazquez et al, 2023; Ridge et al, 2024; Salomonsson et al, 2019). Recently, their numbers have also been found to decrease after treatment with anti–IL-5 and anti–IL-5Ra therapies in allergic asthma (Alvarado-Vazquez et al, 2024). Intriguingly, these cells have also been found to be increased in nonatopic, inflammatory disease states (Lei et al, 2024). Recently, Lin^−^CD117^+^CD34^+^FceRI^+^ progenitor cells predicted treatment response to omalizumab in chronic spontaneous urticaria (Ridge et al, 2024). This study seeks to identify and quantify Lin^−^CD117^+^CD34^+^FceRI^+^ progenitor cells in the peripheral blood of 10 individuals with a clinical diagnosis of AD and 10 healthy controls.

## Results and Discussion

**Figure 1:**
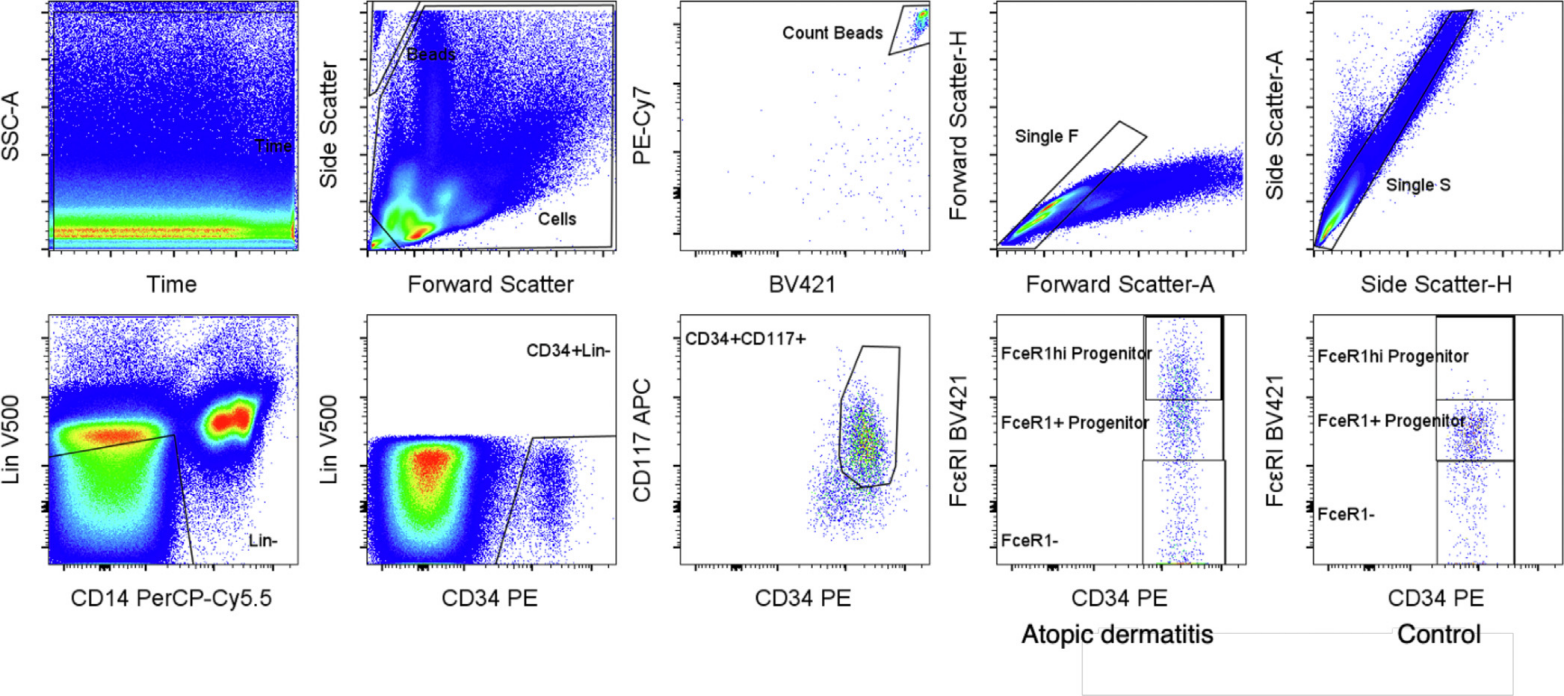
**Gating of Lin^−^CD117^+^CD34^+^ progenitor cells showing FceRI^−^ cells, FceRI^+^ cells, and FceRI^hi^ cells in an atopic dermatitis sample and a healthy control.** APC, allophycocyanin; PE, phycoerythrin; SSC-A, side scatter area.

**Figure 2:**
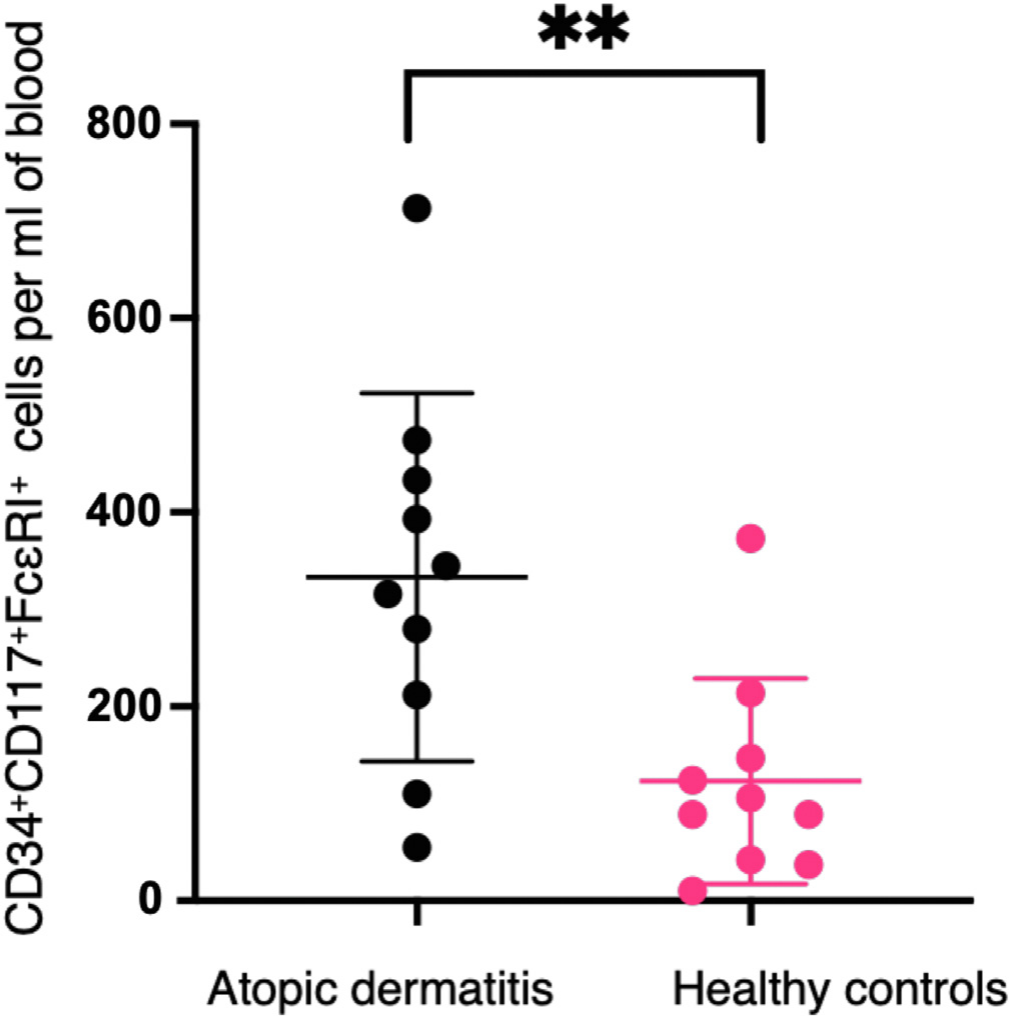
**Lin^−^CD117^+^CD34^+^FceRI^+^ progenitor cells are elevated in atopic dermatitis (n = 10) compared with those in healthy controls (n = 10).***P* = .0067, unpaired *t*-test.

**Figure 3:**
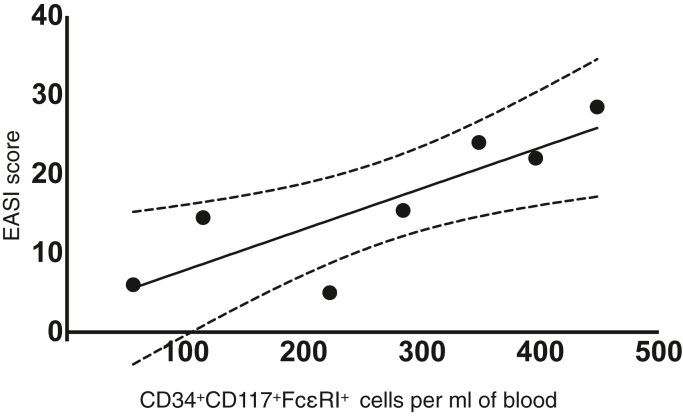
**Lin^−^CD117^+^CD34^+^FceRI^+^ progenitor cells shown with associated EASI score (n = 7).** r = 0.8386. EASI, Eczema Area and Severity Index.

Baseline characteristics are illustrated in Table 1. Figure 1 outlines the gating strategy used to identify cells, with examples of a patient with AD and a healthy control provided. CD34^+^CD117^+^FcεRI^+^ cells were elevated in individuals with AD compared with those in healthy controls (n = 10; *P* = .0067) (Figure 2). There was no difference in FcεRI^−^ or FceRI^hi^ progenitors between individuals with AD and healthy controls (*P* = .356 and *P* = .276, respectively). Mean total serum IgE was not correlated with the number of myeloid progenitors in peripheral blood in individuals with AD (r = 0.486, *P* = .223) or in healthy controls (r = −0.267, *P* = .522). A significant positive correlation was found between FcεRI^+^ progenitors and Eczema Area and Severity Index scores (r = 0.8386, *P* = .0184) (Figure 3). The mean number of circulating FceRI^+^ progenitors in males with AD (n = 6) was 413.3 per ml of peripheral blood compared with 212.8 per ml in females, but this was not statistically significant (*P* = .102). There was no difference in the number of circulating progenitors between male and female controls (*P* = .162).

**Table 1:** Baseline Characteristics

Characteristic	AD (n = 10)	Controls (n = 10)	*P*-Value
Sex	4F:6M	7F:3M	
Age, y, median (range)	22 (18–44)	32 (24–43)	
White cell count (per 10^9^/l), mean (SD)	7.83 (1.94)	6.5 (1.52)	
Eosinophils (per 10^9^/l), mean (SD)	0.76 (0.47)	0.16 (0.1)	
Basophils (per 10^9^/l), mean (SD)	0.05 (0.05)	0.03 (0.05)	
Total serum IgE (kU/l), mean (SD)	3400 (1813)	54.75 (74.86)	
Lin^−^CD34^+^CD117^+^ cells per ml of blood, median (range)	446 (69–1705)	140 (49–501)	.0353^1^
Lin^−^CD117^+^CD34^+^FceRI^+^ cells per ml of blood, median (range)	330.5 (55–713)	97.5 (10–373)	.0067^2^
Disease duration, y, mean (SD)	23.3 (8.64)		
Topical corticosteroids	10/10 (100%)		
Oral corticosteroids within the last 4 wk	0/0		
Previous systemic treatment for AD	0/0		
EASI score^3^, median (range)	15.4 (5–38.5)		
DLQI^4^, median (range)	11 (5–19)		

Abbreviations: AD, atopic dermatitis; DLQI, Dermatology Life Quality Index; EASI, Eczema Area and Severity Index; F, female; M, male.

1 Unpaired *t*-test.

2 Unpaired *t*-test.

3 EASI score interpretation: clear = 0, almost clear = 0.1–1.0, mild = 1.1–7.0, moderate = 7.1–21.0, severe = 21.1–50.0, and very severe = 50.1–72.0.

4 DLQI scores: 0–1 = no effect at all on patient’s life, 2–5 = small effect on patient’s life, 6–10 = moderate effect on patient’s life, 11–20 = very large effect on patient’s life, and 21–30 = extremely large effect on patient’s life.

These data are preliminary and require replication in larger cohorts. However, they are, to our knowledge, a previously unexamined cell type in AD and add to a growing body of evidence on amplification of these cells in atopy. Although mast cells are acknowledged as a primary effector cell in allergic disease, recognition of their function in fields such as autoimmunity and cancer is growing (Maurer et al, 2019; Komi and Redegeld, 2020). Indeed, increased numbers of mast cell progenitors have been reported in the peripheral blood of individuals with rheumatoid arthritis (Lei et al, 2024). Therefore, it is plausible that recruitment of myeloid and mast cell progenitors may not be attributable to an amplification of a type 2 response and that diverse drivers of inflammation may have a role in triggering the egress of these cells. Inflammation may influence leucocyte chemotaxis by altering CXCR4/CXCL12 interactions in the bone marrow (Cambier et al, 2023). Elevated stem cell factor as a result of inflammatory processes may also influence chemotactic gradients and progenitor egress (Nilsson et al, 1994). Contemporaneous tissue quantification of cells may help to clarify whether progenitor cells in peripheral blood reflect increased bone marrow production or reduced emigration into tissues. Identifying the cytokines and transcription factors that incite the release of these cells from the bone marrow are important next steps. Transcriptomic data may help to inform our understanding of these cells, their maturity, and heterogeneity in AD (Ridge et al, 2024).

Findings offer valuable insights into potential differences in circulating myeloid and mast cell progenitors in patients with AD. Notably, an association was observed between FceRI^+^ progenitor cells and clinical severity of AD as assessed by the Eczema Area and Severity Index score. Although the role of mast cells in AD remains to be fully elucidated, it is important to note that the primary pruritogen released by mast cells (histamine) does not represent a major pruritogen in AD, aligning with the limited efficacy of antihistamines in this condition (Rukwied et al, 2000). Nonetheless, the observed difference in FceRI^+^ myeloid progenitors in AD raises the possibility that the bone marrow is responding to chemotactic gradients in AD. Paired analyses of individuals off and on systemic treatment modalities in AD may provide additional insights into the triggers of progenitor egress. Although we did not record a quantitative measure of itch in this cohort, future studies may benefit from interrogating whether clinical measures such as itch correlate with circulating mast cell progenitor burden.

## Materials and Methods

Ethical approval was granted by the Joint Research Ethics Committee in Tallaght University Hospital and St. James’s Hospital in Ireland. Written, informed consent was obtained. Healthy control participants had no diagnosis of atopic disease, which we defined as the absence of self-reported AD, allergic rhinitis, asthma, food allergy, eosinophilic esophagitis, or chronic spontaneous urticaria. We excluded patients with AD who were in receipt of any systemic treatment. Where possible, each patient had an Eczema Area and Severity Index score calculated (Hanifin et al, 2022).

Samples were prepared in line with established protocols (Salomonsson et al, 2019). Whole blood (12–15 ml) was collected in EDTA-treated tubes (BD Vacutainer, BD Bioscience). PBMCs were enriched using Ficoll-Paque Premium (*ρ* = 1.076 g/ml) (GE Healthcare) in SepMate-50 tubes (Stemcell Technologies). PBMCs were incubated in PBS at pH 7.4 with 2% heat-inactivated fetal calf serum with the following fluorescent-labeled antibodies: V500 CD4 (RPA-T4), V500 CD8 (RPA-T8), PerCP-Cy5.5 CD14 (M5E2), V500 CD19 (HIB19), phycoerythrin CD34 (581), allophycocyanin CD117 (104D2), and BV421 FcεRI (AER-37). Total number of single cells and absolute numbers of cells per ml were calculated using BD Biosciences Liquid Counting Beads. Sample acquisition was performed on a FACSCanto II flow cytometer (BD Biosciences). Data analysis was performed using FlowJo (version 10, BD Biosciences). Fluorescence minus one controls were used to confirm CD34, CD117, and FcεRI positivity. We categorized progenitor cells according to whether they were Lin^−^CD34^+^CD117^+^FcεRI^−^ (referred to as FceRI^−^ progenitors), Lin^−^CD34^+^CD117^+^FcεRI^+^ (referred to as FceRI^+^ progenitors), or Lin^−^CD34^+^CD117^+^FcεRI^hi^ (referred to as FceRI^hi^ progenitors) (Figure 1). FceRI^hi^ progenitors represent an extremely rare cell type with the phenotype of true mast cell precursors (Dahlin et al, 2016).

### Statistical analysis

Data were assessed for normality using a Shapiro–Wilk test. Results indicated that variables were normally distributed (W = 0.9638, *P* = .8282). An unpaired *t*-test was used to compare the quantities of progenitors in patients with AD and controls. A Pearson’s r correlation was used to assess for an association between progenitors and Eczema Area and Severity Index scores in patients with AD.

## Ethics Statement

Ethical approval was granted by the Joint Research Ethics Committee in Tallaght University Hospital and St. James’s Hospital in Ireland. Written, informed consent was obtained from all participants.

## Data Availability Statement

Data will be made available upon reasonable request to corresponding author.

## ORCIDs

Katie Ridge: http://orcid.org/0000-0003-4276-7050

Conor M. Finlay: http://orcid.org/0000-0001-8285-0903

Barry Moran: http://orcid.org/0000-0002-8243-1079

Cliona O’Farrelly: http://orcid.org/0000-0002-0616-2874

Julianne Clowry: http://orcid.org/0000-0002-8526-4966

Alan D. Irvine: http://orcid.org/0000-0002-9048-2044

Niall Conlon: http://orcid.org/0000-0002-8023-280X

## Conflict of Interest

NC has received support for attending meetings from Novartis. ADI is a consultant and on the advisory board for AbbVie, Novartis, Regeneron, Sanofi, Leo Pharma, Pfizer, Eli Lilly, Benevolent AI, and Arena. The remaining authors state no conflict of interest.
